# Knowledge and Uses of African Pangolins as a Source of Traditional Medicine in Ghana

**DOI:** 10.1371/journal.pone.0117199

**Published:** 2015-01-20

**Authors:** Maxwell Kwame Boakye, Darren William Pietersen, Antoinette Kotzé, Desiré-Lee Dalton, Raymond Jansen

**Affiliations:** 1 Department of Environmental, Water and Earth Sciences, Tshwane University of Technology, Pretoria, South Africa; 2 National Zoological Gardens of South Africa, Pretoria, South Africa; 3 Genetics Department, University of the Free State, Bloemfontein, South Africa; 4 African Pangolin Working Group, Pretoria, South Africa; Harbin Medical University, CHINA

## Abstract

Traditional medicine has been practised in Ghana for centuries with the majority of Ghanaians still patronising the services of traditional healers. Throughout Africa a large number of people use pangolins as a source of traditional medicine, however, there is a dearth of information on the use of animals in folk medicine in Ghana, in particular the use of pangolins. The aim of this study was to determine the prevalent use of pangolins and the level of knowledge of pangolin use among traditional healers in Ghana for the treatment of human ailments. Data was gathered from 48 traditional healers using semi-structured interviews on the traditional medicinal use of pangolin body parts in the Kumasi metropolis of Ghana. The cultural importance index, relative frequency of citation, informant agreement ratio and use agreement values were calculated to ascertain the most culturally important pangolin body part as well as the level of knowledge dissemination among traditional healers with regards pangolin body parts. Our study revealed that 13 body parts of pangolins are used to treat various medicinal ailments. Pangolin scales and bones were the most prevalent prescribed body parts and indicated the highest cultural significance among traditional healing practices primarily for the treatment of spiritual protection, rheumatism, financial rituals and convulsions. Despite being classified under Schedule 1 of Ghana’s Wildlife Conservation Act of 1971 (LI 685), that prohibits anyone from hunting or being in possession of a pangolin, our results indicated that the use of pangolins for traditional medicinal purposes is widespread among traditional healers in Ghana. A study on the population status and ecology of the three species of African pangolins occurring in Ghana is urgently required in order to determine the impact this harvest for traditional medical purposes has on their respective populations as current levels appear to be unmonitored and unsustainable.

## Introduction

It is currently estimated that 80% of Africa′s population uses traditional medicine (TM) [1] either on its own or collectively with Western medicine [2]. TM plays a very important complementary role in healthcare delivery systems in Ghana as between 70–80% of Ghanaians depend on TM for their primary health care needs [2–4] and therefore it has a high level of cultural acceptability and use. Historically, TM was the only known default form of healthcare until the introduction of modern medicine by the British medical officers during the colonisation of the country [5]. Although the majority of Ghanaians have embraced modern scientific medicine, indigenous health beliefs are commonly sought within urban and rural communities in this country [6] and is recognized by the government as a component of the healthcare delivery system where the Traditional Medicine Practice Act (Act 575 of 2000) provides the legal framework for the practice of TM in Ghana. Compared to other countries in sub-Sahara Africa, Ghana’s TM system is relatively well established because it has been incorporated into the national health programmes and policies [7].

The use of whole, parts or products of animal species for the treatment of a wide range of human ailments in Ghana has been recorded [2, 8, 9] but only Ntiamoa-Baidu [8] mentioned the use of scales of one of the tree pangolin species for the treatment of coughing in Ghana. The use of pangolins in traditional medicinal practices is of particular interest as it is well documented as being used within traditional medicinal systems in Asia [10–13] and as a source of traditional medicine in those parts of Africa where they occur [13–23]. However, the parts of the animal used and the ailments for which they are prescribed are unrecorded. In addition, it is unknown at what level these animals are regarded as an important source of cultural significance within traditional medical practices in Ghana.

There are three species of pangolin found in Ghana; black-bellied pangolin (*Phataginus tetradactyla*), white-bellied pangolin (*Phataginus tricuspis*) and the giant ground pangolin (*Smutsia gigantea*) [24]. As yet, no studies have been conducted investigating offtake or harvest levels, the prevalence of use and level of knowledge of pangolins for medicinal purposes among traditional healers in Ghana. Such information is essential in devising strategies for the conservation of pangolins. Therefore, the aim of this study was to determine the prevalent use of whole animals or body parts and the level of knowledge of pangolins as a medicinal resource among traditional healers in Ghana.

## Materials and Methods

### Study area

The study was conducted in the Kumasi Metropolitan area in the Ashanti Region of Ghana. The Kumasi Metropolitan area is approximate 254 km^2^ and is located between latitudes 6°35” and 6°4”N and longitudes 1°30” and 1°35” E [25]. It shares boundaries with the Kwabre District to the north, Atwima Kwanwoma and Atwima Nwabiagya District to the west, Ejisu-Juaben Municipal to the east and Bosomtwe District to the south. The metropolis has a human population of 2,035,064 constituting 43% of the total human population of 4,780,380 in the Ashanti region [26]. Kumasi is a cosmopolitan city and home to most major ethnic groups from West Africa.

The health services in the municipality are relatively abundant and include the Komfo Anokye Teaching Hospital (KATH), which is one of the two national autonomous hospitals, four quasi health institutions, five health care centres owned by the Church of Christ and the Seventh-Day Adventist Church, over 200 known private health institutions and 13 industrial clinics [27]. By investigating the use of wild animal based remedies in an urban setting, this study attempts to determine the cultural ties between pangolin use as a source of traditional medicine within a metropolitan area that is relatively well equipped with adequate modern healthcare facilities compared to the more rural areas of Ghana.

### Ethics Statement

This study was approved by the ethics committee of the Tshwane University of Technology (REC Ref #: REC2013/05/008) and written informed consent was obtained from all participants interviewed.

### Data collection

Ethnozoological data on medicinal use of pangolins was collected between September 2013 and January 2014 using semi-structured interviews. The sampling process was purposeful with participants intentionally selected because they could provide the relevant information pertinent to this study [28]. Of the 62 traditional healers identified to have used or currently using whole or parts of pangolins in their remedies, 48 agreed to participate in this study. Participants were made aware of their rights to decide to either voluntary participate or to decline. The interview questions focused on the interviewees’ knowledge of the uses of pangolin parts or whole pangolins for medicinal purposes to cure or alleviate a particular medical ailment or a set of ailments. Verbal prompts and probes were used to motivate informants and elicit information from them and the pace as well as direction of the interviews was dictated by the participants. The key questions that were asked to traditional healers (THs) were: (i) do you know the animal called pangolin (participants were shown photographs of African pangolin species) and (ii) do you know whether it is prescribed as a source of medicine? In the semi-structured interviews, the THs (interviewees) were requested to name the parts of pangolins used as medicine, the ailments for which that body part was prescribed and how the animals were obtained. THs were also questioned on how this knowledge was acquired. The interviews were conducted in Twi language which is the most widely spoken language by the Ashanti’s in the metropolis and has also been adopted by many others as a *lingua franca*. MKB conducted the interviews with all the participants where both he all the participants interviewed are fluent in the Twi language.

### Data analysis

Four quantitative value indices were calculated to establish the frequency of use of pangolins or their body parts in TM, as well as the consensus of use body parts among THs: cultural importance index (CI), relative frequency of citation (RFC), informant agreement ratio (IAR), and use agreement value (UAV).

### Cultural Importance Index (CI)

Cultural importance index (CI), developed by Tardio and Pardo-de Santayana [29], takes into account the uniformity of the body part used between THs as well as a body part’s diversity of uses to treat ailments among THs.

$$CI=\sum_{u=u1}^{uNC}\sum_{i=i1}^{iN}\frac{URui}{N}$$

where u is the category of use (e.g.: spiritual ailments, rheumatism, infertility), NC is the total number of different categories of use (of each ‘i’ body part), UR is the total number of use-reports for each body part (corresponding in the present study to ‘citations’, as defined above) and N is the total number of informants.

### Relative Frequency of Citation (RFC)

This index calculates the frequency of use amongst all the TMPs and is obtained by dividing the number of informants who mention the use of the body part (also known as frequency of citation: FC), by the number of informants participating in the survey (N) [29]. Theoretically, the RFC varies from 0 (when no respondents refer to the body part as useful) to 1 (if all the TMPs made use of a particular body part) [29]. The RFC was calculated using the following formula:

$$RFC=\frac{FC}{N}$$

where FC is frequency of citation and N is total number of participants.

### Informant Agreement Ratio (IAR)

The Informant Agreement Ratio (IAR) was used to measure the consensus level for treating a particular ailment making use of a particular pangolin body part amongst THs. The original formula proposed by Trotter and Logan [30] was interpreted as follows:

$$IAR=\frac{nr-na}{nr-1}$$

where *nr* is the total number of use reports registered for a body part and *na* is the number of ailments that are treated with this body part. The IAR value varies between 0 and 1whereby a value of 1 represents the highest level of agreement of a medicinal part.

### Use Agreement Value (UAV)

According to Thomas et al. [31] this index provides a valid and easily derived estimation of the animal’s medicinal cultural significance as a resource. Following the suggestions of Thomas et al. [31], but using UV instead of Quality Use Value (QUV), the use agreement value (UAV) was defined as follows:

UAV=CI×IAR

where CI is the cultural importance index of body part and IAR is the informant agreement ratio of that body part.

## Results

The majority of participants were between the ages of 51–60 and have practised for more than 20 years (Table 1). Regarding gender, the majority were males while Muslims dominated with respect to religion and had a Quranic education. The level of education of the majority of participants was below primary level (Table 1).

**Table 1:** Demographics of participants in the study.

**Gender**	**Number**	**Percentage**
Male	45	94
Female	3	6
**Age group**		
31–40	3	6
41–50	8	17
51–60	21	44
60+	16	33
**Number of years of practice**		
5–10	4	8
11–15	5	10
16–20	8	17
Above 20	31	65
**Last level of education**		
Below primary	30	63
Below secondary	13	27
Secondary	4	8
Post-secondary	1	2
**Religion**		
Muslim	41	85
Christian	6	13
Animist	1	2

A total of 13 pangolin body parts were identified as being used and prescribed for the treatment of 35 ailments (Table 2). Spiritual ailments had the highest use reported (UR) followed (in descending order) by rheumatism, infertility, financial rituals and convulsions (Table 2). Pangolin scales had the highest use report (19 of 35 ailments), followed by bones (14 ailments), the head (10) and meat (9), respectively (Table 2). The heart and limbs had the lowest UR (1).

**Table 2:** Use Report (UR) by traditional healers for particular pangolin body parts for the treatment of specific ailments.

	**Body parts**													**Total**
**Ailments**	**A**	**B**	**C**	**D**	**E**	**F**	**G**	**H**	**I**	**J**	**K**	**L**	**M**	**UR**
spiritual protection	6	2	2	1	3					1			1	16
rheumatism	7	7				1								15
infertility	1		5	1		1		1						9
financial rituals	4		1	1			1			1				8
convulsions	5	1												6
menstrual pains	3			1					1					5
stomach disorders	4										1			5
protection from witchcraft	1	1				1		2						5
headache	1	1	2											4
skin scars	4													4
stroke	1	2	1											4
waist pain		3									1			4
heart disease	2		2											4
back pain	3													3
asthma		1	2											3
mental illness	1	1			1									3
charms for chiefs				3										3
epilepsy	3													3
courting a lady						2								2
bedwetting	1	1												2
waterborne diseases	2													2
fever		1	1											2
gonorrhoea			2											2
preventing miscarriage during pregnancy				1								1		2
treating wounds	2													2
broken leg		1												1
skin rash		1												1
body aches			1											1
scrotal mass									1					
prolonged or continuous menstrual bleeding							1							1
elephantiasis							1							1
impotence					1									1
breast cancer		1												1
leprosy	1													1
coughing				1										1
Total	52	24	19	9	5	5	3	3	2	2	2	1	1	128

A = Scales, B = Bones, C = Head, D = Meat, E = Eyes, F = Tail, G = Whole animal, H = Claws, I = Bile, J = Leg, K = Waist, L = Heart, M = Toes

With regards to the contribution of each of the 13 pangolin body parts to the total cultural importance index (CI), scales had the highest CI followed by the bones, head and meat (Table 3). Spiritual ailments had the highest CI followed (in descending order) by rheumatism, infertility, financial rituals and convulsions. Scales and bones were equally important for the treatment of rheumatism. Scales were also important for spiritual protection and the treatment of convulsions. The head was important for the treatment of infertility while the meat was used for preparing charms for chiefs or tribal leaders. Similar results were obtained when comparing the level of agreement of use of pangolin body parts consistently prescribed by traditional healers for the treatment of particular ailments (i.e. Informant Agreement Ratio, IAR, Table 4). Scales had the highest IAR, followed by the head, bones and meat (Table 4). Scales were predominantly used to treat rheumatism, followed by spiritual protection, convulsions and financial rituals. The head was mainly used to treat infertility while the meat was used in the preparation of charms for chiefs.

**Table 3:** Cultural Importance Index (CI) of pangolin body parts and their respective ailments treated.

	**Body parts**													**Total**
**Ailments**	**A**	**B**	**C**	**D**	**E**	**F**	**G**	**H**	**I**	**J**	**K**	**L**	**M**	**CI**
spiritual protection	0.125	0.042	0.042	0.021	0.063					0.021			0.021	0.333
rheumatism	0.146	0.146				0.021								0.313
infertility	0.021		0.104	0.021		0.021		0.021						0.188
financial rituals	0.083		0.021	0.021			0.021			0.021				0.167
convulsions	0.104	0.021												0.125
menstrual pains	0.063			0.021					0.021					0.104
stomach disorders	0.083										0.021			0.104
protection from witchcraft	0.021	0.021				0.021		0.042						0.104
headache	0.021	0.021	0.042											0.083
skin scars	0.083													0.083
stroke	0.021	0.042	0.021											0.083
waist pain		0.063									0.021			0.083
heart disease	0.042		0.042											0.083
back pain	0.063													0.063
asthma		0.021	0.042											0.063
mental illness	0.021	0.021			0.021									0.063
charms for chiefs				0.063										0.063
epilepsy	0.063													0.063
courting a lady						0.042								0.042
bedwetting	0.021	0.021												0.042
waterborne diseases	0.042													0.042
fever		0.021	0.021											0.042
gonorrhoea			0.042											0.042
preventing miscarriage during pregnancy				0.021								0.021		0.042
treating wounds	0.042													0.042
broken leg		0.021												0.021
skin rash		0.021												0.021
body aches			0.021											0.021
scrotal mass									0.021					0.021
prolonged or continuous menstrual bleeding							0.021							0.021
elephantiasis							0.021							0.021
impotence					0.021									0.021
breast cancer		0.021												0.021
leprosy	0.021													0.021
coughing				0.021										0.021
Total	1.083	0.500	0.396	0.188	0.104	0.104	0.063	0.063	0.042	0.042	0.042	0.021	0.021	2.667

A = Scales, B = Bones, C = Head, D = Meat, E = Eyes, F = Tail, G = Whole animal, H = Claws, I = Bile, J = Leg, K = Waist, L = Heart, M = Toes

**Table 4:** Informant Agreement Ratio (IAR) of pangolin body parts and their respective ailments treated.

	**Body parts**							**Total**						
**Ailments**	**A**	**B**	**C**	**D**	**E**	**F**	**G**	**H**	**I**	**J**	**K**	**L**	**M**	**IAR**
spiritual protection	0.075	0.036	0.053	0.028	0.300					<0.000			<0.000	0.491
rheumatism	0.087	0.127				0.050								0.264
infertility	0.012		0.132	0.028		0.050		0.167						0.388
financial rituals	0.050		0.026	0.028			<0.000			<0.000				0.104
convulsions	0.062	0.018												0.080
menstrual pains	0.037			0.028					<0.000					0.065
stomach disorders	0.050										<0.000			0.050
protection from witchcraft	0.012	0.018				0.050		0.333						0.414
headache	0.012	0.018	0.053											0.083
skin scars	0.050													0.050
stroke	0.012	0.036	0.026											0.075
waist pain		0.054									<0.000			0.054
heart disease	0.025		0.053											0.078
back pain	0.037													0.037
asthma		0.018	0.053											0.071
mental illness	0.012	0.018			0.100									0.131
charms for chiefs				0.083										0.083
epilepsy	0.037													0.037
courting a lady						0.100								0.100
bedwetting	0.012	0.018												0.031
waterborne diseases	0.025													0.025
fever		0.018	0.026											0.044
gonorrhoea			0.053											0.053
preventing miscarriage during pregnancy				0.028								<0.000		0.028
treating wounds	0.025													0.025
broken leg		0.018												0.018
skin rash		0.018												0.018
body aches			0.026											0.026
scrotal mass									<0.000					<0.000
prolonged or continuous menstrual bleeding							<0.000							<0.000
elephantiasis							<0.000							<0.000
impotence					0.100									0.100
breast cancer		0.018												0.018
leprosy	0.012													0.012
coughing				0.028										0.028
Total	0.647	0.435	0.500	0.250	0.500	0.250	0.000	0.500	<0.000	<0.000	<0.000	<0.000	<0.000	3.082

A = Scales, B = Bones, C = Head, D = Meat, E = Eyes, F = Tail, G = Whole animal, H = Claws, I = Bile, J = Leg, K = Waist, L = Heart, M = Toes

Appreciable differences were observed when comparing the various indices with one another (Table 5). With the exception of scales which ranked in first position for all four indices, the order varied for the other body parts depending on the index chosen. The CI, RFC and UAV indices place bones and the head in second and third positions, respectively. This was not the same for IAR, which ranked the head above the bones. The heart and limbs were the lowest in all indices.

**Table 5:** Evaluation of pangolin body parts using Cultural Importance Index (CI), Relative Frequency of Citation (RFC), Informant Agreement Ratio (IAR) and Use Agreement Value (UAV).

	**Basic Value**			**Indices**				**Ranking**			
**Body part**	**UR**	**FC**	**NU**	**CI**	**RFC**	**IAR**	**UAV**	**CI**	**RFC**	**IAR**	**UAV**
scales	52	29	19	1.083	0.604	0.647	0.701	1	1	1	1
bones	24	15	14	0.500	0.313	0.435	0.217	2	2	3	2
head	19	14	10	0.396	0.292	0.500	0.198	3	3	2	3
meat	9	9	7	0.188	0.188	0.250	0.047	4	4	4	5
eye	5	5	3	0.104	0.104	0.500	0.052	5	5	2	4
tail	5	5	4	0.104	0.104	0.250	0.026	5	5	4	7
whole animal	3	2	3	0.063	0.042	<0.000	<0.000	6	7	5	8
claws	3	3	2	0.063	0.063	0.500	0.031	6	6	2	6
bile	2	1	2	0.042	0.021	<0.000	<0.000	7	8	5	8
leg	2	1	2	0.042	0.021	<0.000	<0.000	7	8	5	8
waist	2	1	2	0.042	0.021	<0.000	<0.000	7	8	5	8
heart	1	1	1	0.021	0.021	<0.000	<0.000	8	8	5	8
toes	1	1	1	0.021	0.021	<0.000	<0.000	8	8	5	8

UR = use report, FC = frequency of citation, NU = number of uses

## Discussion

### Cultural importance index (CI)

Pangolin scales are highly valued in African traditional medicine pharmacopoeia where they are widely used. Our study confirmed this in that we found knowledge about pangolin scales used for medicinal purposes to be more widespread than any other pangolin body part. Our findings agree with previous studies in Africa in that scales are the most versatile pangolin body part for the treatment of a variety of ailments [14, 19, 20, 23]. Indeed, the suite of ailments treated by pangolin scales in this study was similar to the ailments treated in other African countries [14, 18, 19, 20, 23]. Not only were scales prescribed for physical medical ailments but they were also prescribed to prevent or inhibit spiritual ailments as was found to be the case in previous studies [18, 19, 20, 23].

The relatively high CI index for pangolin bones also suggests that bones have an important role to play in traditional healing practices and previous studies have also indicated that they are prescribed for the treatment of rheumatism, stroke and pains in Nigeria [19, 20] and Sierra Leone [23]. Our study, however, found the versatility of pangolin bones for the treatment of more than one ailment to be greater in Ghana than was indicated for Nigeria [19, 20] and Sierra Leone [23]. Pangolin heads have previously been recorded being used to treat fever in Benin Republic in West Africa [18] and for treating spiritual ailments in Nigeria [19, 20]. We also found the head to be very important in the treatment of infertility, headache and heart diseases which has also been found to be the case in Sierra Leone [23].

Bräutigam et al. [14] found pangolin meat to be less valued for medicinal purposes compared to other body parts, however our study indicates that among the 13 pangolin body parts used by traditional healers, the meat was the fourth most culturally important after scales, the head and bones. However, knowledge about the therapeutic abilities of the meat was less widespread and was mostly limited to the preparation of charms for chiefs. The eyes were primarily prescribed for spiritual protection but were also used to treat kleptomania as was found in Nigeria [19, 20] and Apollo (acute haemorrhagic conjunctivitis) in Sierra Leone [23]. The pangolin heart had a very low index value and was occasionally used to prevent miscarriage in Ghana although Akpona et al. [18] documented the use of pangolin heart and leg to treat tachycardia, and promote normal growth and vigour in babies in Benin Republic. The low use value and medicinal application of the bile, leg, heart, waist, claws and toes are indications that these pangolin body parts are culturally less important to the traditional healers of Ghana than other body parts.

### Informant Agreement Ratio (IAR)

The high IAR for scales and bones is an indication that these items are important components of local cultural knowledge within the traditional healer community [32] and an indication that a particular pangolin part is preferred in the community for treatment of specific ailments [33–35]. On the contrary, the low IAR for the whole animal, bile, leg, waist, heart and toes may be an indication that these body parts have either fallen into disuse because of cultural adaptation or are believed to be ineffective for treating conditions or ailments or may simply be of a low cultural importance in traditional medicine. Culturally bound syndromes are usually bound to yield high consensus values due to their folk nature [36] with traditional healers often preferring a particular body part for the treatment of a particular ailment because belief systems do not usually permit the prescription of a substitute resource as a form of medical treatment. We found culturally bound syndromes such as spiritual ailments, financial rituals, protection from witchcraft, preparation of charms for chiefs as well as courting ladies scoring high IAR for particular body parts used. For instance, there may not be any other substitute part for the pangolin meat which was the only body part used in the preparation of charms for chiefs.

One disease that was surprisingly not mentioned but that is widespread in the study area and impacts upon a large number of local communities is malaria. The selection and use of a resource for traditional medicinal purposes are usually based on culturally perceived effectiveness [36] that is generally handed down from generation to generation. The perceived ineffectiveness of pangolin body parts for the treatment of malaria may have accounted for its noticeable absence in the list of diseases mentioned by traditional healers.

### Comparing different indices

With the exception of scales, which occupied the top ranking for all indices, the other body parts varied in their ranking in the different indices. The distribution of knowledge about traditional remedies follows a pattern whereby few remedies are known to almost everyone while most knowledge is idiosyncratic [31] and this idiosyncrasy may account for the variation in indices for body parts, particularly for the IAR.

Training to become a traditional medical practitioner within Ghana is either via family tradition or an informal apprenticeship [3, 5]. Trainees undergo a period of training during which he/she learns the curative potential of plants, animals and minerals through the observation of their “master healer” [5, 37]. Those traditional healers that partook in this study were all trained via a family tradition and obtained their knowledge verbally from either a mother, father, uncle or grandparent and therefore this knowledge is likely to be idiosyncratic in nature and, in turn, would influence the results and the consensus on use of a particular pangolin body part.

Thomas et al. [31] established that the combination of the number of use reports and the level of consensus between participants seems to provide a valid and easily derived estimate of cultural significance and it can therefore be deduced that the culturally important pangolin body parts found in this study, such as scales, bones and head, are those with high use agreement values. It is therefore proposed that pangolins have been utilised for some time as a constant and frequent source of traditional medicine and it plays a large culturally important role in Ghanaian traditional pharmacopoeia.

## Conservation Implications

All three species of African pangolin that occur within Ghana; the white-bellied pangolin (*Phataginus tricuspis*), black-bellied pangolin (*Phataginus tetradactyla*) and the giant ground pangolin (*Smutsia gigantea*) have been up-listed to *Vulnerable* on the recently revised IUCN Red List [38]. In addition, the Wildlife Conservation Act 1971 (LI 685) classifies them as Schedule 1 and this prohibits their hunting or possession. Despite their conservation and protection status, pangolins are still being hunted for their purported medicinal values and the law regarding the protection of the species is not enforced. Based on the CI, IAR and UAV indices, where pangolin body parts are used to treat ailments by traditional healers, this study has found that the use of pangolins in local community pharmacopoeia is quite common. However, if this use and harvest is unsustainable and off-take exceeds recruitment into the population, it can have drastic consequences on their natural population status [39–41]. With such apparent frequent use, it is of concern that the harvest of these endangered mammals is unsustainable, particularly when most of the trade in pangolins is informal. Furthermore, our results indicate that pangolins are used to treat folk illnesses which usually have no substitute or alternative remedies with healers relying on what they are culturally familiar with. This use of pangolins to treat folk illnesses, such as protection against witchcraft, spiritual protection and the courting of ladies, can be considered an additional threat to the level of harvesting of pangolins in Ghana as these illnesses are considered to be intrinsic to the Ghanaian cultural concepts of health and illness [5, 42]. The rate of harvest is further increased by the high CI of pangolin body parts for ailments that have been found to be medically or socially prevalent in Ghana. Rheumatism, which has a high CI, has been listed among the top 10 diseases seen at outpatient departments in hospitals and clinics across Ghana [43], and convulsions are also common due to Ghana being a malaria hyper-endemic country [44, 45]. Again, infertility has a high CI index for pangolin body parts and is considered a social problem in the country and the belief in superstitions is most often ‘cured’ or remedied via traditional healers prescribing pangolin body parts [46]. The fact that most of the body parts used by traditional healers can only be obtained after killing the animal raises further concerns about the impact that harvesting pressure has on pangolins. Furthermore, there are no *ex situ* breeding facilities for African pangolins, thus all individuals that are used for medicinal purposes are being obtained from the wild [19, 20]. Even though the healing properties and effectiveness of pangolin body parts have not been scientifically tested, they remain of large cultural significance due to their purported healing properties and the harvest of these species will continue unabated. These cultural medical knowledge systems are often organised within local cultural communities [47] and the belief system of TMPs may not offer a substitute for a particular remedy thus making sole reliance on pangolin body parts inevitable. A tribe or community’s cultural and traditional background and belief systems rather than pharmacological properties often plays a greater role in determining the effectiveness of traditional medicine [48].

The impact that traditional medicine is having on the survival and conservation status of wild pangolin populations should be considered as an important population threat to these mammals just as other anthropogenic pressures may be [21, 49]. This form of harvest on wild pangolin populations should be carefully assessed and monitored as current turn-over rates in the utilisation of these species for traditional medicine is not known and very little data exists on current population levels and distribution of these three species in Ghana [50]. Furthermore, little information exists on the levels of trade and harvest of biodiversity from the wild that is used for traditional medicine in Ghana [51]. It is therefore important that we increase our understanding of the biology, ecology and population status of species commonly used as traditional remedies to better assess the impact of harvesting on wild populations [52]. As such, conservation strategies for pangolins in Ghana would first need to focus on understanding the current population status, biology and ecology of the species in order to effectively assess the effect of this harvest on wild populations. In the meantime, conservation efforts should be aimed at educating traditional healers on the implications of unsustainable harvest of wild pangolins and the potential threat that the loss of these species may have on local community cultural belief systems if they are no longer available.

## Conclusion

The cultural belief system is most likely to be the driving force for the maintenance and continued use of pangolins in urban environments for medicinal purposes. Popular knowledge about the curative properties of pangolin body parts is an integral part of the local culture and demonstrates the necessity of carefully studying the use of pangolins for therapeutic practices to better understand the human cultural interaction. There is a need to undertake multidisciplinary studies to investigate the social, cultural, and economic aspects of pangolin use in traditional medicinal practices in Africa in order to develop sound conservation management strategies and action plans for these species.

## References

[1] WHO (2002) WHO Traditional medicine strategy 2002–2005. Geneva Switzerland WHO/EDM/TRM/2002.

[2] Dove N (2010) A return to traditional health care practices: a Ghanaian study. Journal of Black Studies 40(5): 823–834. 10.1177/0021934708320001

[3] Abel C, Busia K (2005) An exploratory ethnobotanical study of the practice of herbal medicine by the Akan Peoples of Ghana. Alternative Medicine Review 10(2): 112–122. 15989380

[4] Asante E, Avornyo R (2013) Enhancing healthcare system in Ghana through integration of traditional medicine. Journal of Sociological Research 4(2): 256–272.

[5] Twumasi PA (1979) History of pluralistic medical systems: a sociological analysis of the Ghanaian case. Journal of Opinion 9(3): 29–34. 10.2307/1166260

[6] Tabi MM, Powell M, Hodnicki D (2006) Use of traditional healers and modern medicine in Ghana. International Nursing Review 53: 52–58. 10.1111/j.1466-7657.2006.00444.x 16430761

[7] Sato A (2012) Rationales for traditional medicines utilisation and its equity implications: the case of Ghana. Ph.D. Thesis, The London School of Economics and Political Science. Available: http://etheses.lse.ac.uk/491/1/sato_Rationales%20for%20Traditional%20Medicines%20utilisation%20and%20its%20Equity%20Implications.pdf. Accessed 2014 Sep 1.

[8] Ntiamoa-Baidu Y (1997) Wildlife and food security in Africa. FAO Conservation Guide 33. Available: http://www.fao.org/docrep/w7540e/w7540e00.htm. Accessed 2014 Jul 10.

[9] Insoll T (2011) Substance and materiality? The archaeology of Talensi medicine shrines and medicinal practices. Anthropology & Medicine 18(2): 181–203. 10.1080/13648470.2011.591196 21810036 PMC3379785

[10] Gaski AL, Johnson KA (1994) Prescription for extinction: endangered species and patented oriental medicines in trade. TRAFFIC Network Report, TRAFFIC USA.

[11] Kang S, Phipps M (2003) A question of attitude: South Korea’s traditional medicine practitioners and wildlife conservation. TRAFFIC East Asia, Hong Kong.

[12] Kaspal P, Shah KB, Singh SK, Baral HS (2012) Community-based conservation initiative of Chinese pangolin *Manis pentadactyla* in the community forests of Bhaktapur, Central Nepal. In: Katuwal HB, Koirala S, editors. Proceedings of third seminar on small mammals issues. Small Mammals Conservation and Research Foundation, New Baneshwor, Kathmandu, Nepal, pp. 37–42.

[13] Chakkaravarthy QA (2012) Research and conservation needs of the Indian pangolin (*Manis crassicaudata*). In: Katuwal HB, Koirala S, editors. Proceedings of third seminar on small mammals issues. Small Mammals Conservation and Research Foundation, New Baneshwor, Kathmandu, Nepal, pp. 50–55.

[14] Bräutigam A, Howes J, Humphreys T, Hutton J (1994) Recent information on the status and utilization of African pangolins. TRAFFIC Bulletin 15(1): 15–22.

[15] Sodeinde OA, Adedipe SR (1994) Pangolins in south-west Nigeria—current status and prognosis. Oryx 28(1): 43–50. 10.1017/S0030605300028283

[16] Marshall NT (1998) Searching for a cure: conservation of medicinal wildlife resources in east and southern Africa. TRAFFIC International. A TRAFFIC Network Report.

[17] Sodeinde OA, Soewu DA (1999) Pilot study of the traditional medicine trade in Nigeria. TRAFFIC Bulletin 18(1): 35–40.

[18] Akpona HA, Djagoun CAMS, Sinsin B (2008) Ecology and ethnozoology of the three-cusped pangolin *Manis tricuspis* (Mammalia, Pholidota) in the Lama forest reserve, Benin. Mammalia 72: 198–202. 10.1515/MAMM.2008.046

[19] Soewu DA, Ayodele IA (2009) Utilisation of pangolin (*Manis sps*) in traditional Yorubic medicine in Ijebu province, Ogun State, Nigeria. Journal of Ethnobiology and Ethnomedicine 5: 39–49. 10.1186/1746-4269-5-39 19961597 PMC2797502

[20] Soewu DA, Adekanola TA (2011) Traditional-medical knowledge and perception of pangolins (*Manis sps*) among the Awori people, Southwestern Nigeria. Journal of Ethnobiology and Ethnomedicine 7: 25–35. 10.1186/1746-4269-7-25 21884607 PMC3179697

[21] Challender DWS, Hywood L (2012) African pangolins: under increased pressure from poaching and intercontinental trade. TRAFFIC Bulletin 24(2): 53–55.

[22] Soewu DA, Bakare OK, Ayodele IA (2012) Trade in wild mammalian species for traditional medicine in Ogun State, Nigeria. Global Journal of Medical Research 12(3): 6–22.

[23] Boakye MK, Pietersen DW, Kotzé A, Dalton DL, Jansen R (2014) Ethnomedicinal use of African pangolins by traditional medical practitioners in Sierra Leone. Journal of Ethnobiology and Ethnomedicine 10: 76–86. 10.1186/1746-4269-10-76 25412571 PMC4247607

[24] Gaudin TJ, Emry RJ, Wible JR (2009) The phylogeny of living and extinct pangolins (Mammalia, Pholidota) and associated taxa: A morphology based analysis. Journal of Mammalian Evolution 16: 235–305. 10.1007/s10914-009-9119-9

[25] Ghana Districts (2006) A repository of all districts in the republic of Ghana. Available: http://www.ghanadistricts.com/districts/?news&r=2&_=6. Accessed 2014 May 28.

[26] Ghana Statistical Service (2012) Population by region, district, age groups and sex, 2010. Available: http://www.statsghana.gov.gh/docfiles/pop_by_region_district_age_groups_and_sex_2010.pdf. Accessed 30 May 2014.

[27] Kumasi Metropolitan Assembly (2006). About this metropolis. Available: http://www.kma.ghanadistricts.gov.gh/?arrow=atd&_=6&sa=1823. Accessed 2014 Jun 2.

[28] Babbie ER (2001) The practice of social research. Cape Town: Oxford University Press 674p.

[29] Tardio J, Pardo-de Santayana M (2008) Cultural importance indices: a comparative analysis based on the useful wild plants of southern Cantabria (northern Spain). Economic Botany 62: 24–39. 10.1007/s12231-007-9004-5

[30] Trotter RT, Logan MH (1986) Informant consensus: a new approach for identifying potentially effective medicinal plants. In: Etkin NL, editor. Plants in indigenous medicine and diet. Redgrave Publishing Company, Bedford Hill, New York, pp. 91–112.

[31] Thomas E, Vandebroek I, Sanca S, Van Dammem P (2009) Cultural significance of medicinal plant families and species among Quechua farmers in Apillapampa, Bolivia. Journal of Ethnopharmacology 122: 60–67. 10.1016/j.jep.2008.11.021 19101618

[32] Heinrich M, Edwards S, Moerman DE, Leonti M (2009) Ethnopharmacological field studies: a critical assessment of their conceptual basis and methods. Journal of Ethnopharmacology 124: 1–17. 10.1016/j.jep.2009.03.043 19537298

[33] Gazzaneo LRS, Lucena RFP, Albuquerque UP (2005) Knowledge and use of medicinal plants by local specialists in an region of Atlantic Forest in the state of Pernambuco (Northeastern Brazil). Journal of Ethnobiology and Ethnomedicine 1: 9–24. 10.1186/1746-4269-1-9 16270911 PMC1291389

[34] Almeida CFCBR, Amorim ELC, Albuquerque UP, Maia MBS (2006) Medicinal plants popularly used in the Xingó region—a semi-arid location in Northeastern Brazil. Journal of Ethnobiology and Ethnomedicine 2: 15–21. 10.1186/1746-4269-2-15 16556305 PMC1444943

[35] Alves RRN, Rosa IL (2006) From cnidarians to mammals: the use of animals as remedies in fishing communities in NE Brazil. Journal of Ethnopharmacology 107: 259–276. 10.1016/j.jep.2006.03.007 16621379

[36] Heinrich M, Ankli A, Frei B, Weimann C, Sticher O (1998) Medicinal plants in Mexico: healers′ consensus and cultural importance. Social Science & Medicine 47(11): 1859–1871. 10.1016/S0277-9536(98)00181-6 9877354

[37] Omolewa M (2007) Traditional African modes of education: their relevance in the modern world. International Review of Education 53: 593–612. 10.1007/s11159-007-9060-1

[38] Challender DWS, Waterman C, Baillie JEM (2014) Scaling up pangolin conservation. IUCN SSC Pangolin Specialist Group Conservation Action Plan. Zoological Society of London, London, UK Available: http://www.pangolinsg.org/files/2012/07/Scaling_up_pangolin_conservation_280714_v4.pdf. Accessed 2014 Aug 8.

[39] Alves RRN, Rosa IL (2005) Why study the use of animal products in traditional medicines? Journal of Ethnobiology and Ethnomedicine 1: 5–9. 10.1186/1746-4269-1-5 16270931 PMC1277085

[40] Alves RRN, Santana GG (2008) Use and commercialization of *Podocnemis expansa* (Schweiger 1812) (Testudines: Podocnemididae) for medicinal purposes in two communities in North of Brazil. Journal of Ethnobiology and Ethnomedicine 4: 3–8. 10.1186/1746-4269-4-3 18208597 PMC2254592

[41] Alves RRN, Lima HN, Tavares MC, Souto WMS, Barboza RRD, et al (2008) Animal-based remedies as complementary medicines in Santa Cruz do Capibaribe, Brazil. BMC Complementary and Alternative Medicine 8: 44–52. 10.1186/1472-6882-8-44 18647413 PMC2503950

[42] de-Graft Aikins A, Anum A, Agyemang C, Addo J, Ogedegbe O (2012) Lay representations of chronic diseases in Ghana: implications for primary prevention. Ghana Medical Journal 46(2): 59–68. 23661819 PMC3645147

[43] Ghana Health Service (2010) Annual Report. Available: http://www.ghanahealthservice.org/includes/upload/publications/GHS_2010_Annual%20Report_Final.pdf. Accessed 2014 Jul 15.

[44] Department for International Development (DFID) (2011) Malaria: Country Profiles. Version 1.1. Available: https://www.gov.uk/government/…data/…/malaria-country-profiles.pdf. Accessed 2014 May 15.

[45] President’s Malaria Initiative (2012) Malaria Operational Plan—FY 2012 (Year 5) Ghana. Available: www.pmi.gov/…/malaria-operational-plans/…/ghana_mop_fy12.pdf?…4. Accessed 2014 May 15.

[46] Tabong PT-N, Adongo PB (2013) Understanding the social meaning of infertility and childbearing: a qualitative study of the perception of childbearing and childlessness in northern Ghana. PLoS ONE 8(1): e54429 10.1371/journal.pone.0054429 23342158 PMC3546987

[47] Costa-Neto EM (2004) Implications and applications of folk zootherapy in the state of Bahia, northeastern Brazil. Sustainable Development 12(3): 161–174. 10.1002/sd.234

[48] Moerman DE (2007) Agreement and meaning: rethinking consensus analysis. Journal of Ethnopharmacology 112: 451–460. 10.1016/j.jep.2007.04.001 17524581

[49] Pietersen DW, McKechnie AE, Jansen R (2014) A review of the anthropogenic threats faced by Temminck’s ground pangolin, *Smutsia temminckii*, in southern Africa. South African Journal of Wildlife Research 44(2): 167–178. 10.3957/056.044.0209

[50] Challender D, Gabriel GG, Pietersen D, Jansen R, Hywood L (2014) Protecting pangolins. Asian Geographic 103(2): 86–91.

[51] Ministry of Health (2005) Policy guidelines on traditional medicine development. Available: www.moh-hana.org/…/TRADITIONAL%20MEDICINE%20POLICY_2. Accessed 2014 Aug 20.

[52] Alves RRN, Rosa IL, Santana GG (2007) The role of animal-derived remedies as complementary medicine in Brazil. BioScience 57(11): 949–955. 10.1641/B571107

